# The fragmentomic property of plasma cell-free DNA enables the non-invasive detection of diabetic nephropathy in patients with diabetes mellitus

**DOI:** 10.3389/fendo.2023.1164822

**Published:** 2023-10-05

**Authors:** Chaolun Yu, Yu Lin, Yuxue Luo, Yun Guo, Zhiming Ye, Rijing Ou, Yan Zhang, Xinxin Wang, Ruokai Qu, Wenwen Zhou, Jie Li, Yong Bai, Xueqing Yu, Haiqiang Zhang, Li Yan, Xin Jin

**Affiliations:** ^1^ Department of Endocrinology, Sun Yat-sen Memorial Hospital, Sun Yat-sen University, Guangzhou, China; ^2^ Guang Dong Clinical Research Center for Metabolic Diseases, Sun Yat-sen Memorial Hospital, Sun Yat-sen University, Guangzhou, China; ^3^ BGI Research, Shenzhen, China; ^4^ School of Medicine, South China University of Technology, Guangzhou, Guangdong, China; ^5^ The First Clinical Medical College, Guangdong Medical University, Zhanjiang, China; ^6^ Department of Nephrology, Guangdong Provincial People’s Hospital, Guangdong Academy of Medical Sciences, Guangzhou, China; ^7^ Guangdong-Hong Kong Joint Laboratory on Immunological and Genetic Kidney Diseases, Guangdong Provincial People’s Hospital, Guangdong Academy of Medical Sciences, Guangzhou, China; ^8^ School of Biology and Biological Engineering, South China University of Technology, Guangzhou, China

**Keywords:** cfDNA, liquid biopsy, kidney disease, endocrine disease, biomarkers

## Abstract

**Background:**

Diabetic nephropathy (DN) is one of the most prevalent complications of diabetes mellitus (DM). However, there is still a lack of effective methods for non-invasive diagnosis of DN in clinical practice. We aimed to explore biomarkers from plasma cell-free DNA as a surrogate of renal biopsy for the differentiation of DN patients from patients with DM.

**Materials and methods:**

The plasma cell-free DNA (cfDNA) was sequenced from 53 healthy individuals, 53 patients with DM but without DN, and 71 patients with both DM and DN. Multidimensional features of plasma DNA were analyzed to dissect the cfDNA profile in the DM and DN patients and identify DN-specific cfDNA features. Finally, a classification model was constructed by integrating all informative cfDNA features to demonstrate the clinical utility in DN detection.

**Results:**

In comparison with the DM patients, the DN individuals exhibited significantly increased cfDNA concentration in plasma. The cfDNA from the DN patients showed a distinct fragmentation pattern with an altered size profile and preferred motifs that start with “CC” in the cfDNA ending sites, which were associated with deoxyribonuclease 1 like 3 (*DNASE1L3*) expression in the kidney. Moreover, patients with DM or DN were found to carry more alterations in whole-genome cfDNA coverage when compared with healthy individuals. We integrated DN-specific cfDNA features (cfDNA concentration, size, and motif) into a classification model, which achieved an area under the receiver operating characteristic curve (AUC) of 0.928 for the differentiation of DN patients from DM patients.

**Conclusion:**

Our findings showed plasma cfDNA as a reliable non-invasive biomarker for differentiating DN patients from DM patients. The utility of cfDNA in clinical practice in large prospective cohorts is warranted.

## 1 Introduction

Diabetic nephropathy (DN), a major complication of diabetes and one of the leading causes of chronic kidney disease (CKD) and end-stage renal disease (ESRD) (1–3), is characterized by albuminuria and a reduced glomerular filtration rate (GFR), accompanied by glomerular and tubulointerstitial histological damage (4, 5). It has been reported that 20% to 50% of patients suffering from diabetes may eventually develop DN 10–20 years after the onset of diabetes (6).

Renal biopsy is the gold standard for the diagnosis of DN and is also a reliable method for distinguishing patients with non-diabetic renal disease (NDRD) from those with non-specific DN (7). However, the invasiveness, the procedural cost, and the potential risk for the development of complications during renal biopsy hinder the wide use of this technique clinically. In current practice, serum creatinine (8), estimated GFR (eGFR), and microalbuminuria (MA) (9) are widely adopted to assess the progression of DN. However, their specificity and sensitivity for the precise diagnosis of DN are limited. Thus, the development of novel non-invasive methods for the accurate detection of DN is needed.

Circulating cell-free DNA (cfDNA) has been applied in non-invasive prenatal testing (NIPT) and cancer detection. The alteration of cfDNA fragment size (10), methylation patterns (11–14), somatic mutations, and copy number variations (12, 15) were pivotal characteristics of cfDNA for disease diagnosis or detection. In recent years, cfDNA has also attracted much attention as a novel non-invasive biomarker in kidney diseases (16). However, the lack of comprehensive consideration of cfDNA features in previous studies hampers the development of cfDNA-based diagnostic biomarkers with high sensitivity and specificity.

We aimed to comprehensively investigate the properties of plasma cfDNA related to DN and build a classification model by integrating the identified cfDNA features to differentiate DN patients from diabetes mellitus (DM) patients.

## 2 Materials and methods

### 2.1 Patients and samples

A total of 177 participants were recruited for this study, including 71 DM patients with biopsy-proven DN, 53 patients with type 2 DM only, and 53 healthy subjects aged between 18 and 75 years from the Medical Laboratory Center, Endocrinology Department, and Nephrology Department at Guangdong Provincial People’s Hospital between August 2019 to August 2021. All the DM patients were diagnosed more than 8 years ago and had a urine albumin/creatinine ratio (UACR) ≤30 mg/g or an eGFR ≥ 60 ml·min^−1^·1.73 m^−2^. eGFR was calculated by the CKD-EPIcr (ml·min^−1^·1.73 m^−2^) formula (17). The DN patients were further classified into five stages according to the eGFR values; more specifically, the DN patients with eGFR >90, 60–90, 30–60, 15–30, and <15 were assigned to stages 1, 2, 3, 4, and 5, respectively. Patients with cancer, acute infection, immunosuppressant usage, and pregnancy were excluded. The detailed clinical information of all the subjects is presented in **Table 1**. The study was approved by the ethics committee of Guangdong Provincial People’s Hospital (Approval No. GDREC2019771H(R1)) and the Institute Review Board of BGI (BGI-IRB 20111-T1). Informed consent forms were signed by all participants.

**Table 1:** Demographic and clinical characteristics of control, DM, and DN subjects in this study.

Variables	DN^a^	DM^b^	Control^c^	p-Value	*Post hoc*
	n = 71	n = 53	n = 53		
Age (year)	55.07 ± 7.67	58.67 ± 8.55	43.11 ± 8.59	<0.001	a, b > c
Gender (M/F)	54/17	28/25	24/29	0.001	
BMI (kg/m^2^)	24.84 ± 2.72	24.27 ± 3.46	23.26 ± 3.39	0.04	a > c
Hypertension (%)	87%	25%	12%	<0.001	
Systolic blood pressure (mmHg)	149.03 ± 20.53	134.6 ± 18.20	127.15 ± 16.28	<0.001	a > b, c
Diastolic blood pressure (mmHg)	81.89 ± 10.15	77.50 ± 9.06	77.56 ± 11.62	0.04	a > c
Cardio-cerebrovascular disease (%)	39%	53%	0%	<0.001	
Diabetic retinopathy (%)	69%	None	None		
DM duration (month)	104.75 ± 90.69	173.90 ± 72.30	None	<0.001	b > a
Fasting glucose (mmol/L)	6.41 ± 3.71	7.62 ± 3.20	4.67 ± 0.46	<0.001	b > a > c
HbA1C (%)	6.74 ± 1.64	8.24 ± 1.95	5.31 ± 0.42	<0.001	b > a > c
Serum creatinine (μmol/L)	303.55 ± 214.42	71.80 ± 15.91	73.85 ± 15.74	<0.001	a > b, c
Uric acid (μmol/L)	437.21 ± 115.62	381.99 ± 94.31	377.53 ± 127.76	0.009	a > b, c
Urine A/C ratio (mg/g)	3671.21 ± 2839.19	15.40 ± 18.81	7.96 ± 3.33	<0.001	a > b, c
eGFR (ml/(min * 1.73 m^2^))	33.69 ± 26.96	92.32 ± 17.13	98.98 ± 15.23	<0.001	b, c > a
HDL (mmol/L)	1.10 ± 0.31	1.28 ± 0.32	1.43 ± 0.42	<0.001	a > b > c
LDL (mmol/L)	3.58 ± 1.62	3.50 ± 1.28	3.71 ± 1.10	0.78	
TG (mmol/L)	2.11 ± 1.43	1.74 ± 1.14	2.00 ± 3.07	0.70	
Cholesterol (mmol/L)	5.54 ± 2.36	5.54 ± 1.72	5.97 ± 1.64	0.46	
WBC (10^9^/L)	7.77 ± 1.72	6.74 ± 1.25	6.53 ± 1.41	<0.001	

The p-value of the measurement data was calculated by one-way ANOVA with Bonferroni for multiple comparisons; if the homogeneity of variance was not satisfied, p-value was from Kruskal–Wallis test alternatively. Type I error of 0.05 level. The categorical variables such as gender, hypertension, and cardio-cerebrovascular disease were compared by a chi-square test.

Urine A/C ratio, urine albumin-creatinine ratio; BMI, body mass index; DM, diabetes mellitus; eGFR, estimated glomerular filtration rate; HDL, high-density lipoprotein; LDL, low-density lipoprotein; TG, triglycerides; WBC, white blood cell.

### 2.2 cfDNA extraction and library preparation

The peripheral blood sample of each subject was collected in EDTA tubes. Within 4 hours of blood collection, blood samples were centrifuged at 1,600 × *g* for 10 min at 4°C. The supernatant was isolated and recentrifuged for 10 min at 16,000 × *g* at 4°C. Then, the plasma was separated and stored at −80°C for further experiments. A MagPure Circulating DNA KF Kit (Magen, Guangzhou, China) was used to extract cell-free DNA from 200 µl of plasma. Sequencing libraries were prepared and amplified through 12 cycles of PCR using an MGIEasy Cell-free DNA Library Prep kit (MGI) according to the manufacturer’s instructions. cfDNA concentration was measured using a Qubit dsDNA HS Assay Kit (Q32854, Invitrogen, Carlsbad, CA, USA) with a Qubit™ 3 Fluorometer (Invitrogen) in plasma and amplified libraries before sequencing.

### 2.3 DNA sequencing and data alignment

DNA libraries were sequenced with a paired-end format of 100 bp × 2 on the DNBSEQ platform (MGI, Shenzhen, China). At least 100 Gb of raw sequencing data was obtained for each sample. Adaptor sequences and low-quality bases were removed from the raw reads by using fastp (v0.20.1) (18). The clean reads in FASTQ format were then aligned to the human reference genome (GRCh38/hg38) using minimap2 (v2-2.11) (19), and PCR duplicates were filtered with biobambam2 (v2-2.0.87) (20). Reads aligning to multiple locations of the genome or with mismatches of more than 3 were further filtered. Paired-end reads that aligned with corrected strands and orientations with an insert size within 600 bp were retained for downstream analysis.

### 2.4 Motif analysis of plasma DNA

As previously defined (21), the ends motif in this study refers to the first four nucleotides at the 5′ end of each strand of plasma DNA molecules. The frequency of each type of 256 motifs was calculated to assess the motif occurrence. The motif diversity score described in the previous study (21) was adopted to measure the uniformity of motif distribution. High motif diversity score (MDS) represented a uniform distribution of ends motif in frequencies, whereas a low MDS represented a skewed distribution. The motif ratio was calculated by the following formula:


$$\text{Motif}\_\text{ratio}\_\left(\text{n}\right)=\frac{\sum_{i=1}^{n}\text{Motif}\_\text{up}\left(i\right)}{\sum_{i=1}^{n}\text{Motif}\_\text{down}\left(i\right)},$$

where Motif_up(*i*) and Motif_down(*i*) indicate the *i*th significantly increased and decreased motifs in the DN patients compared with the DN patients, respectively. The 4-mer motif frequency and MDS were further calculated in DNA fragments across different sizes.

### 2.5 Analysis of measured genomic representations

The DNA fraction in each of the 1-Mb non-overlapping bins across the whole genome in plasma samples was calculated, termed as the measured genomic representation (MGR) of this bin. As previously described (22), we adopted the z-score to quantify the normalized deviations of MGR from the reference group consisting of 10 randomly selected control samples in each bin. The MGR-based scores were explored in all the DM and DN samples, as well as control samples except those used for building reference. Those bins with a z-score <−3 or >3 were identified as bins with aberrant MGR. The MGR z-scores across the whole genome were visualized by a circos plot (23).

### 2.6 XGBoost-based prediction model for DN diagnosis

A total of 124 samples (71 DN and 53 DM) were first split into a training dataset and an independent testing dataset in an 8:2 ratio by adopting the randomly stratified sampling method for preserving the same proportions of DN and DM in the two datasets. The training dataset (57 DN and 42 DM) was used for training the XGBoost-based classification model (24), and its hyperparameters were tuned through randomized search with fivefold cross-validation. To mitigate the negative effect of the imbalanced dataset, the class weight for each sample during the training phase was additionally calculated. The classification performance was then evaluated using the independent testing dataset (14 DN and 11 DM) via 100 iterations of bootstrapping (25) sampling with replacement. For the classification of early DN patients, 15 DN patients were randomly selected at stages 1–3 and 20 DM samples as the testing set. The remaining samples were used for training the classification model and its hyperparameters with the same method mentioned above. The 100 iterations of bootstrapping sampling with replacement were also applied to assess the performance of early DN classification in the testing dataset (15 early DN and 20 DM).

### 2.7 Statistical analysis

In the analysis of each cfDNA feature, observations deviating from the mean ± 4*standard deviations were considered outliers and removed in that feature analysis to eliminate potential bias. The Mann–Whitney U-test (Wilcoxon rank sum tests) and Kruskal–Wallis test were applied for the evaluation of differences in continuous variables in different groups. The chi-square test or Fisher’s exact test was employed for the comparison of categorical variables. Spearman’s test was used to assess the correlation. For all informative cfDNA features showing significant differences between the DM and DN groups, multivariable logistic regression was performed to examine the associations between cfDNA characteristics and disease outcome with adjustment for age, gender, and body mass index (BMI). All the receiver operating characteristic (ROC) analyses in this study were also adjusted for the same covariates. p-Values below 0.05 were considered statistically significant. p-Values less than 0.05, 0.01, 0.001, and 0.0001 were represented by the symbols *, **, ***, and ****, respectively. R software (version 3.6.1) program was used for statistical analysis.

## 3 Results

### 3.1 Patient characteristics

We recruited 53 healthy individuals with neither DM nor DN, 53 patients with DM but without DN, and 71 patients with both DM and DN in this study (see Materials and Methods). The demographic and clinical characteristics of all the subjects are presented in **Table 1**. The median age (55.07 *vs.* 58.67 years, p = 0.053) and BMI (24.84 *vs.* 24.27 kg/m^2^, p = 0.64) were comparable between the DN and DM groups. However, the DN cohort had a significantly higher proportion of male patients than the DM group (76.06% *vs.* 52.83%, p-value = 0.012). Compared with the DN and DM patients, the control subjects showed a younger age (median, 43.11 years), lower BMI (median, 23.26 kg/m^2^), and lower proportion of male patients (median, 45.28%). The majority of the DN patients had hypertension (87%), while this proportion was lower in the DM (25%) and control (12%) groups. As all the DN patients in this study were confirmed by renal biopsy, which was routinely guided by clinical indicators, the serum creatinine, uric acid, urine A/C ratio, and eGFR in the DN patients were significantly different from those in the DM patients.

### 3.2 Cell-free nuclear DNA and mitochondrial DNA level in plasma of DN patients

**Figure 1:**
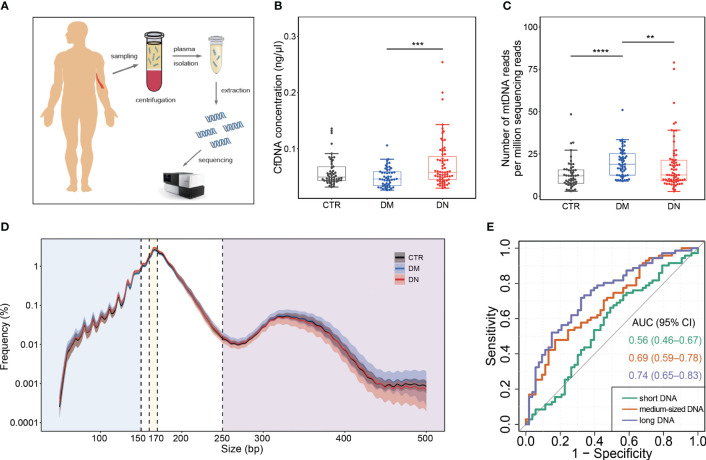
Plasma DNA collection and analysis of basic cfDNA features. **(A)** Sample collection and analysis. **(B)** The concentration of cfDNA in plasma. **(C)** The abundance of mtDNA reads among all sequencing reads. **(D)** Size profiles of plasma DNA from control, DM, and DN subjects on a logarithmic scale. The median size profiles for control, DM, and DN subjects are shown as black, blue, and red lines, respectively. The shadow flanking the median line indicates the range of standard deviation. The blue, yellow, and purple regions separated by vertical dash lines represent the regions of small size (≤150 bp), medium size (160–170 bp), and large size (≥250 bp). **(E)** ROC curves on the use of cfDNA fraction in different size intervals for discriminating the DN patients from the DM patients. cfDNA, cell-free DNA; DM, diabetes mellitus; DN, diabetic nephropathy; ROC, receiver operating characteristic. **p <= 0.01 ; ***p <= 0.001 ; ****p <= 0.0001.

Whole-genome sequencing of circulating cfDNA was performed on plasma samples of all the subjects (**Figure 1A**). A median of 941.28 million (range, 418.56–1,597.68 million) uniquely mapped paired-end reads was obtained from each sample.

As shown in **Figure 1B**, the cfDNA concentration increased significantly in plasma samples of the DN patients (median, 0.07 ng/μl; range, 0.03–0.25 ng/μl) compared with the DM patients (median, 0.05 ng/μl; range, 0.027–0.11 ng/μl) (p-value < 0.001). Although the number of mitochondrial DNA reads per million sequencing reads in the DM patients (median, 18.90, range, 8.98–50.97) was significantly higher than that in the healthy individuals (median, 12.04; range, 2.97–48.40) (p-value < 0.001) and the DN patients (median, 12.57; range, 2.86–79.00) (p-value = 0.006; **Figure 1C**), after correcting age, gender, and BMI, the association between mtDNA abundance and DN/DM outcomes became insignificant (**Supplementary Table 1**).

### 3.3 Size profiles of plasma DNA in DN patients

The size distributions of plasma DNA from the control, DM, and DN patients are shown in **Figure 1D**. A predominant peak at 166 bp was observed in all groups of plasma samples, which is reminiscent of the DNA length wrapping the intact nucleosome (26). In comparison with the healthy individuals and DM patients, the DN patients exhibited a reduction of long DNA. To have an in-depth quantification of the difference between the DN and DM groups, we separated the DNA molecules into three size groups, specifically, those shorter than 150 bp (i.e., short DNA), those between 160 bp and 170 bp (i.e., medium-sized DNA), and those longer than 250 bp (i.e., long DNA). There was no significant difference between the DN and DM patients for the fraction of short DNA (p-value = 0.16) (**Supplementary Figure 1A**). In contrast, the DN patients (median, 38.50%; range, 32.16%–47.96%) showed a significantly higher proportion of medium-sized DNA than the healthy individuals (median, 36.17%; range, 31.52%–43.35%) (p-value < 0.001) and DM patients (median, 37.18%; range, 28.18%–46.29%) (p-value = 0.003; **Supplementary Figure 1B**, **Supplementary Table 1**). Furthermore, a dramatic reduction of long DNA was observed in the DN patients (median, 4.01%; range, 1.49%–8.21%) compared with the DM patients (median, 5.24%; range, 0.84%–10.44%) (p-value < 0.001) **Supplementary Figure 1C**, **Supplementary Table 1**). The area under the ROC curve (AUC) was 0.74 for the DN identification based on the proportion of long DNA, which was higher than the use of short DNA (AUC, 0.56) and medium-sized DNA (AUC, 0.69) (**Figure 1E**).

### 3.4 Diversity of ends motif in plasma DNA of DN patients

**Figure 2:**
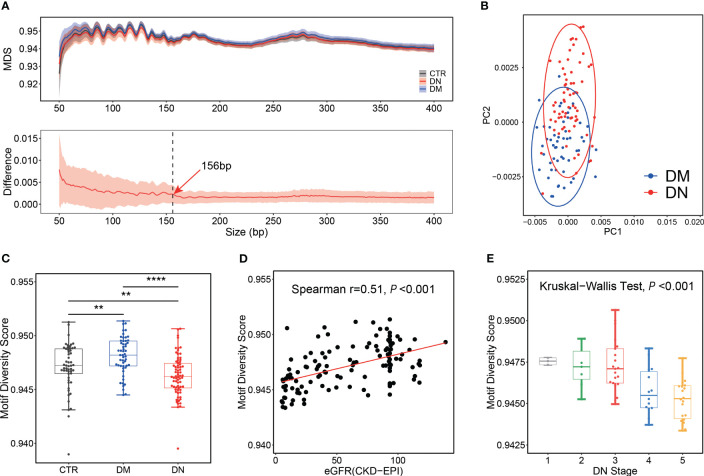
Motif analysis for control, DM, and DN subjects. **(A)** MDS distribution pattern. Top panel: distributions of MDS in plasma DNA across different sizes. The median MDS profiles for control, DM, and DN subjects are shown as black, blue, and red lines, respectively. Bottom panel: distribution of the MDS difference between DN and DM samples across different sizes. The red line indicates the median of differences between the MDS of each DN sample and the average MDS in the DM group. The shadow flanking of the MDS line indicates its standard deviation. The dashed line marks the size of 156 bp. **(B)** PCA on the frequencies of all 256 motifs of short cfDNA (≤156 bp) between DM and DN patients. **(C)** Box plot of motif diversity score in plasma DNA with short length among different groups. **(D)** Correlation between eGFR and MDS of short plasma DNA in DM and DN samples. **(E)** MDS of short plasma DNA in DN samples across different stages determined by eGFR. DM, diabetes mellitus; DN, diabetic nephropathy; MDS, motif diversity score; PCA, principal component analysis; eGFR, estimated glomerular filtration rate. **p <= 0.01 ; ****p <= 0.0001.

MDS (21) calculated from normalized Shannon entropy was adopted to compare the distribution pattern of 256 4-mer motifs in the ending site of plasma DNA in the DN and DM patients (see Materials and Methods). The median MDS of plasma DNA ends in the DN patients and controls was 0.9434 and 0.9463, respectively, which was significantly lower than in the DM patients (median, 0.9472; p-value <0.001 and 0.02; **Supplementary Figure 2A**). The same decrease in MDS of the DN patients was observed across different plasma DNA sizes (top panel of **Figure 2A**). All three groups exhibited similar fluctuations of MDS in DNA size from 50 bp to 400 bp, with highly periodic peaks in small size regions (e.g., size less than ~150 bp). We found that the difference in MDS values between the DM and DN samples was higher and showed periodicity below 156 bp, while the difference values above 156 bp kept dropping and eventually flattened (bottom panel of **Figure 2A**). Hence, we compared the motif signatures of plasma DNA in short (i.e., ≤156 bp) and large sizes (i.e., >156 bp) between the DN patients and other groups. As presented in **Figure 2B**, principal component analysis (PCA) based on the frequencies of all 256 types of ends motifs of short plasma DNA showed a clear separation between the DN and DM patients, while the PCA was not able to separate these two groups based on plasma DNA without size selection (**Supplementary Figure 2B**). Moreover, we found that the MDS of plasma DNA with a small size showed the most significant difference (p-value = 3e−7) between the DN and DM patients compared with that of all DNA (p-value = 2e−6) and long DNA (p-value = 3e−5) (**Figure 2C**, **Supplementary Figures 2A, C**). Moreover, with the use of short DNA, we achieved a higher AUC of 0.77 in discriminating the DN and DM patients compared with the use of long DNA (AUC = 0.72) and all DNA (AUC = 0.75) (**Supplementary Figure 2D**). These results indicated that the selection of short DNA is indeed conducive to the differentiation of DM and DN patients based on motif features.

To further test whether this end motif diversity could reflect the clinical status of the DM and DN patients, we explored the correlation between MDS of short plasma DNA and eGFR. As shown in **Figure 2D**, the MDS value appeared to be positively correlated with eGFR in the DM and DN patients (Spearman’s r = 0.51; p-value < 0.001). Meanwhile, the MDS of plasma DNA progressively decreased as the stage of DN determined by eGFR increased (Kruskal–Wallis p-value < 0.001; **Figure 2E**). These findings greatly proved the clinical values of ends motif diversity of plasma DNA in non-invasively detecting and monitoring DN patients.

### 3.5 Diagnostic value of ends motifs associated with DN

**Figure 3:**
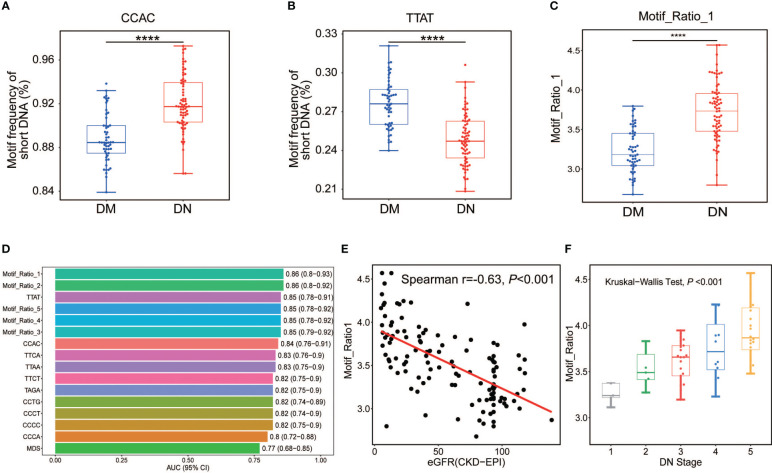
Analysis of DN-specific motifs in short cfDNA. Top 1 motif with highly increased **(A)** and decreased **(B)** frequencies in plasma DNA of DN patients compared with those in DM patients. **(C)** Ratio between the frequencies of top 1 increased motif (CCAC) and top 1 decreased motif (TTAT) in DN and DM patients. **(D)** AUC values of all informative motif features in the determination of DN and DM patients. **(E)** Correlation between eGFR and top 1 motif ratio of plasma DNA in DM and DN samples. **(F)** Top 1 motif ratio of plasma DNA in DN samples across different stages determined by eGFR. DN, diabetic nephropathy; cfDNA, cell-free DNA; DM, diabetes mellitus; AUC, area under the receiver operating characteristic curve; eGFR, estimated glomerular filtration rate. ****p <= 0.0001.

As the diversity of cfDNA ends motifs exhibited distinct patterns in the DN and DM patients, we wondered whether particular motifs could be directly used as diagnostic biomarkers in DN identification. We, therefore, compared each type of ends motifs of plasma short DNA in terms of frequency between the DN and DM patients. CCAC was identified as the most increased motif in the DN patients, showing a relative increase of 4.55% in the median motif frequency (**Figure 3A**). In contrast, TTAT was identified as the most decreased motif in the DN patients, with a relative reduction of 10.71% (**Figure 3B**). Other motifs among the top 5 increased motifs in the DN patients included CCCC, CCCT, CCTG, and CCCA. The top 5 decreased motifs also included TTCA, TTAA, TTCT, and TAGA (**Supplementary Figure 3** and **Supplementary Table 1**). **Figure 3C** shows the ratio between CCAC and TTAT in the DN and DM patients. The AUC values ranged from 0.80 to 0.85 for these top motifs (**Figure 3D** and **Supplementary Table 2**). Among them, the TTAT motif achieved the best performance with an AUC of 0.85. The motif ratios (see Materials and Methods) (AUC range, 0.85–0.86) showed a robust and enhanced performance on DN determination compared with using single motifs (AUC range, 0.80–0.85). Among them, the top 1 motif ratio (ratio between CCAC and TTAT motif) exhibited the highest AUC value of 0.86 (0.80–0.92) (**Figure 3D**). We also observed a strong negative correlation between the motif_ratio_1 and eGFR value in the DM and DN patients (Spearman’s r = −0.63, p-value < 0.001; **Figure 3E**). Moreover, the motif_ratio_1 was found to increase across the DN stages (Kruskal–Wallis test p < 0.001; **Figure 3F**), further consolidating the clinical values of cfDNA ends motif in the diagnosis of DN in patients suffering from DM.

### 3.6 Measured genomic representations of plasma DNA in DN patients

**Figure 4:**
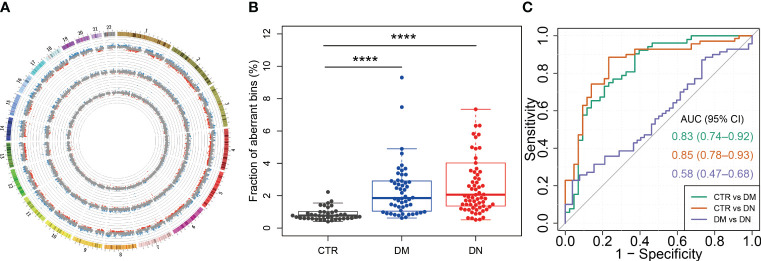
cfDNA distribution across the genome in DM and DN patients. **(A)** Genome-wide distribution of cfDNA for a representative case from each of the control, DM, and DN groups. The inner, middle, and outer rings show the data of cfDNA distribution from a control case (H62), a DM case (M07), and a DN case (K60), respectively. Each dot shows the z-score of a 1-Mb bin. The blue and red dots indicate that the cfDNA in this bin is significantly overrepresented (i.e., z-score > 3) and underrepresented (i.e., z-score < −3), respectively. The scale line in gray around each ring denotes a z-score difference of 5. **(B)** Fraction of bins with overrepresented (i.e., z-score > 3) and underrepresented (i.e., z-score < −3) cfDNA in control, DM, and DN subjects. **(C)** ROC curves with the use of MGR fraction for the determination of DM and DN patients from control subjects, as well as the classification between DM and DN patients. cfDNA, cell-free DNA; DM, diabetes mellitus; DN, diabetic nephropathy; ROC, receiver operating characteristic; MGR, measured genomic representation. ****p <= 0.0001.

To assess the cfDNA distribution patterns in healthy individuals and patients with DM or DN, we surveyed the measured genomic representation of cfDNA across the whole genome (see Materials and Methods) as previously described (22). **Figure 4A** shows the MGR patterns of one representative case from the healthy individuals, DM, and DN groups. The density of bins with aberrant MGR was progressively increased from the control group to the DN group (**Figure 4A**, the innermost to the outermost ring of the circos plot). Percentages of bins with aberrant MGR were significantly higher in the DM (median, 1.85%; range, 0.62%–9.30%) (p-value < 0.0001) and DN groups (median, 2.07%; range, 0.51%–7.85%) (p-value < 0.0001) than the control group (median, 0.78%; range, 0.40%–2.16%; **Figure 4B**). Based on these aberrant MGRs of plasma DNA, the AUC for the identification of the DM and DN patients from controls reached 0.83 and 0.85, respectively. However, the abnormal MGRs were not able to differentiate DN from the DM patients (AUC = 0.58) (**Figure 4C**). These findings revealed the drastic alterations in plasma DNA distribution in patients with DM and DN diseases.

### 3.7 Classification model for DN and DM based on the integration of cfDNA features

**Figure 5:**
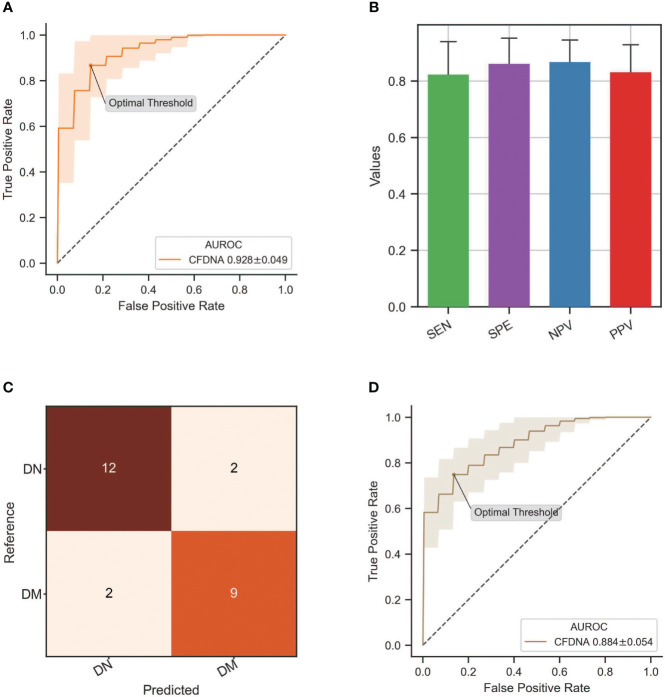
Performance of DN classification by integrating all informative cfDNA features. **(A)** ROC curve of the XGBoost-based model integrating all cfDNA features for DN detection. **(B)** Sensitivity, specificity, negative predictive value (NPV), and positive predictive value (PPV) in the DN identification by using an XGBoost-based classification model. **(C)** The classification results from the testing dataset with the optimal threshold. **(D)** ROC curve of the XGBoost-based model in the differentiation of DN patients at stages 1–3 from DM patients. DN, diabetic nephropathy; cfDNA, cell-free DNA; ROC, receiver operating characteristic; DM, diabetes mellitus.

Since particular cfDNA characteristics displayed diagnostic values in DN identification, we attempted to establish an XGBoost-based classification model (see Materials and Methods) to enhance the DN diagnosis by using all informative cfDNA features, including the proportion of medium-sized (160–170 bp) and long (>250 bp) cfDNA, frequencies of top 5 increased and decreased motifs of short DNA (≤156 bp) in the DN group, top 1 to top 5 motif ratios, and MDS of short cfDNA. As a result, we obtained an AUC value of 0.928 ± 0.049 in the differentiation of DN samples from DM patients (**Figure 5A**), with a sensitivity of 0.821 ± 0.120, a specificity of 0.859 ± 0.094, a negative predictive value of 0.865 ± 0.081, and a positive predictive value of 0.829 ± 0.100 (**Figure 5B**). With the optimal threshold determined by Youden’s J statistic, we detected 12 out of 14 DN samples while misclassifying two out of 11 DM samples (**Figure 5C**). Moreover, we also evaluated the performance of these cfDNA features in the identification of DN at early stages (stages 1–3) determined by eGFR and achieved an AUC of 0.884 ± 0.054 (**Figure 5D**), which demonstrated the feasibility of cfDNA biomarkers in the early detection of DN patients.

## 4 Discussion

In this study, we investigated the comprehensive characteristics of plasma DNA in DM patients complicated with or without DN in a single-base resolution and genome-wide manner. We observed aberrant concentration, size profile, ends motif pattern, and genome distribution of plasma cfDNA in the DN patients. The cfDNA biomarkers we developed in this research achieved a high AUC of 0.928 in the identification of DN patients and presented great potential to be a promising non-invasive surrogate of renal biopsy with high sensitivity and specificity.

The distribution pattern of the ends motifs in the DN and DM patients was observed to be distinct, showing a lower diversity in DN patients. The most preferred ends motif in DN patients started with “CC”. Chan et al. (27) and Jiang et al. (21) have demonstrated that the occurrence of motifs starting with “CC” in plasma DNA ends is closely associated with the activity of the nuclease of *DNASE1L3*. Therefore, we speculated that in DN patients, kidney damage due to DN progression might lead to a more intense process of cell death potentially from the diabetic kidney, which resulted in the increase of kidney-released cell-free DNA that carries the kidney-specific digestion signals in the plasma of DN patients. To validate this hypothesis, we analyzed the RNA-seq data of paired blood cells (the predominant origin of cfDNA in plasma) and kidney tissues from 18 individuals from the Genotype-Tissue Expression (GTEx) database (28). Indeed, the expression level measured by FPKM (fragments per kilobase of exon per million mapped fragments) of *DNASE1L3* gene in kidney tissue was found to be 40-fold higher than that in the blood cell (median, 15.42 *vs.* 0.38) (p-value < 0.001) (**Supplementary Figure 4**). Hence, the altered cfDNA end motifs in DN patients may be majorly attributed to the cellular nuclease activity exhibited in plasma. This finding provides insights into the mechanisms related to the generation of cfDNA in the plasma of DN patients.

Moreover, the motif diversity and informative motifs exhibited strong correlations with the level of eGFR (Spearman’s r = 0.51 and −0.63, respectively), which enabled us to differentiate the DM patients with DN from those without DN. Notably, we found the short DNA (≤150 bp) carried stronger DN signatures in the ends motif, which further improved the diagnostic performance of cfDNA in DN determination. This finding indicated that the cfDNA with different lengths might be generated from different digestion patterns, whereas the short cfDNA may carry more abundant DN signals and were more likely to be released by the kidney tissue.

Another noteworthy finding is that both the DM and DN patients showed altered cfDNA distribution patterns in the genome compared with the healthy individuals. In a previous study, Chan et al. (22) proved that the aberrations in plasma DNA distribution were partly attributed to DNA binding by immunoglobulin G (IgG) class antibodies. Meanwhile, the concentration of immunoglobulin G (IgG) in blood has been reported to be elevated in patients with type 1 diabetes (29). Whether the unevenness of plasma DNA across the human genome is related to the increased concentration of IgG antibodies in the blood of patients with the onset and progression of diabetes is worth exploring in-depth in the future.

Clinically, microalbuminuria and albuminuria are the most common markers used to monitor kidney function and predict the occurrence of diabetic kidney disease. However, their sensitivity and specificity are limited. Therefore, renal biopsy is still essential for DN diagnosis and assessment in the clinic setting, but its invasiveness limits the use to effectively monitor the DN progression by multiple sampling. The cfDNA biomarkers we developed in this research achieved high AUCs of 0.93 and 0.88 in the differentiation of DN patients and those at an early stage from DM patients, which presented great potential to be a promising non-invasive surrogate to solve this predicament with high sensitivity and specificity.

A major limitation of our study was, first, the small sample size of 71 DM patients and 53 DN patients. Thus, a larger cohort collected from multiple centers is required to validate the results further and strengthen the classification model. Second, our study lacked follow-up samples, which limits the exploration of our cfDNA markers in the detection of DN prior to clinical diagnosis in DM patients. It would be of great value if multi-point sampling could be included in future studies to further extend the clinical applications of these cfDNA biomarkers identified from this study.

In summary, we demonstrated the clinical values of cfDNA as a promising non-invasive biomarker for the detection of DN from DM patients through its comprehensive characteristics. Furthermore, the cfDNA profile in plasma provided important insights into the mechanisms underlying the generation and release of cfDNA from DN patients. Thus, the use of plasma cell-free DNA may enable us to detect the onset of DN from the DM cohort in a fast and low-cost manner, which may have great benefits to the early detection and intervention of DN, thereby effectively preventing or delaying the DN progression to the ESRD.

## Data availability statement

The datasets presented in this study can be found in online repositories. The names of the repository/repositories and accession number(s) can be found below: https://db.cngb.org/cnsa/, CNP0002950.

## Ethics statement

The studies involving humans were approved by the Ethics committee of Guangdong Provincial People’s Hospital (Approval No. GDREC2019771H(R1)) and the Institute Review Board of BGI (BGI-IRB 20111-T1). The studies were conducted in accordance with the local legislation and institutional requirements. The participants provided their written informed consent to participate in this study.

## Author contributions

Conceptualization: YL, YB, XY, HZ, LY, and XJ. Methodology: CY, YL, YXL, YG, JL, YB, XY, HZ, LY, and XJ. Software: YL, YB, and HZ. Formal analysis: CY, YL, YXL, and HZ. Investigation: CY, YL, YXL, RO, YZ, XW, ZY, RQ, and WZ. Resources: ZY, XY, HZ, YL, and XJ. Data curation: CY, YL, YXL, and HZ. Writing—original draft: CY, YL, YXL, YB, and HZ. Writing—review and editing: XY, LY, HZ, and XJ. Visualization: YL and HZ. Project administration: XY, HZ, LY, and XJ. Funding acquisition: XY and XJ. All authors contributed to the article and approved the submitted version.

## Supplementary material

Supplementary Figure 1
**(A)** Box plot of the fraction of cfDNA with small size, **(B)** medium size and **(C)** large size in control, DM and DN subjects.

Supplementary Figure 2 Motif diversity score of plasma DNA with different sizes. **(A)** MDS of plasma DNA with all size. **(B)** PCA analysis on the frequencies of all 256 motifs of cfDNA without size selection. **(C)** MDS of plasma DNA with large size. **(D)** ROC analysis of MDS of plasma DNA with different sizes in the identification of DN patients from DM patients.

Supplementary Figure 3 Distribution of DN-specific motifs in control, DM and DN groups.

Supplementary Figure 4 Expression levels of DNASE1L3 gene in whole blood cells (WBC) and kidney tissue, which were calculated by the transcriptome data of paired WBC samples and kidney tissue samples of 18 adults from the GTEx database.

Supplementary Table 1 P-values of the association between cfDNA features and DM/DN outcomes in logistic regression with taking age, gender and BMI as covariates. All the motif related features are based on short cfDNA (<=156bp).

Supplementary Table 2 AUC values of all informative motif features in the determination of DN and DM patients.

## References

[1] Gross JL De Azevedo MJ Silveiro SP Canani LH Caramori ML Zelmanovitz T. Diabetic nephropathy: Diagnosis, prevention, and treatment. Diabetes Care (2005) 28(1):164–76. doi: 10.2337/diacare.28.1.164 15616252

[2] Geerlings SE Hoepelman AIM. Immune dysfunction in patients with diabetes mellitus (DM). FEMS Immunol Med Microbiol (1999) 26(3-4):259–65. doi: 10.1016/S0928-8244(99)00142-X 10575137

[3] Dunea G. Oxford textbook of clinical nephrology. JAMA J Am Med Assoc (1992) 268(17):2441–2441. doi: 10.1001/jama.1992.03490170113045

[4] Morrish NJ Wang SL Stevens LK Fuller JH Keen H. Mortality and causes of death in the WHO multinational study of vascular disease in diabetes. Diabetologia (2001) 44:S14–21. doi: 10.1007/PL00002934 11587045

[5] Cole JB Florez JC. Genetics of diabetes mellitus and diabetes complications. Nat Rev Nephrol (2020) 16(7):377–90. doi: 10.1038/s41581-020-0278-5 PMC9639302 32398868

[6] Sagoo MK Gnudi L. “Diabetic nephropathy: an overview.,”. Methods Mol Biol (2020), 3–7. doi: 10.1007/978-1-4939-9841-8_1 31701441

[7] Arora P Roychaudhury A Pandey R. Non-diabetic renal diseases in patients with diabetes mellitus clinicopathological correlation. Indian J Nephrol (2020) 30(5):295. doi: 10.4103/ijn.IJN_13_19 33707815 PMC7869641

[8] Levey AS Eckardt KU Tsukamoto Y Levin A Coresh J Rossert J. Definition and classification of chronic kidney disease: A position statement from Kidney Disease: Improving Global Outcomes (KDIGO)z. Kidney Int (2005) 67(6):2089–100. doi: 10.1111/j.1523-1755.2005.00365.x 15882252

[9] Warram JH Scott LJ Hanna LS Wantman M Cohen SE Laffel LMB. Progression of microalbuminuria to proteinuria in type 1 diabetes: Nonlinear relationship with hyperglycemia. Diabetes (2000) 49(1):94–100. doi: 10.2337/diabetes.49.1.94 10615955

[10] Yamamoto Y Uemura M Nakano K Hayashi Y Wang C Ishizuya Y. Increased level and fragmentation of plasma circulating cell-free DNA are diagnostic and prognostic markers for renal cell carcinoma. Oncotarget (2018) 9(29):20467. doi: 10.18632/oncotarget.24943 29755665 PMC5945531

[11] Cheng THT Jiang P Tam JCW Sun X Lee WS Yu SCY. Genomewide bisulfite sequencing reveals the origin and time-dependent fragmentation of urinary cfDNA. Clin Biochem (2017) 50(9):496–501. doi: 10.1016/j.clinbiochem.2017.02.017 28238813

[12] Lasseter K Nassar AH Hamieh L Berchuck JE Nuzzo PV Korthauer K. Plasma cell-free DNA variant analysis compared with methylated DNA analysis in renal cell carcinoma. Genet Med (2020) 22(8):1366–73. doi: 10.1038/s41436-020-0801-x 32341571

[13] Li W Zhou XJ. Methylation extends the reach of liquid biopsy in cancer detection. Nat Rev Clin Oncol (2020) 17(11):655–6. doi: 10.1038/s41571-020-0420-0 PMC8284425 32732909

[14] Nuzzo PV Berchuck JE Korthauer K Spisak S Nassar AH Abou Alaiwi S. Detection of renal cell carcinoma using plasma and urine cell-free DNA methylomes. Nat Med (2020) 26(7):1041–3. doi: 10.1038/s41591-020-0933-1 PMC8288043 32572266

[15] Ge G Peng D Guan B Zhou Y Gong Y Shi Y. Urothelial carcinoma detection based on copy number profiles of urinary cell-free DNA by shallow whole-genome sequencing. Clin Chem (2020) 66(1): 188–98. doi: 10.1373/clinchem.2019.309633 31811000

[16] Perego RA Corizzato M Brambilla P Ferrero S Bianchi C Fasoli E. Concentration and microsatellite status of plasma DNA for monitoring patients with renal carcinoma. Eur J Cancer (2008) 44(7):1039–47. doi: 10.1016/j.ejca.2008.03.008 18397824

[17] Vistisen D Andersen GS Hulman A Persson F Rossing P Jørgensen ME. Progressive decline in estimated glomerular filtration rate in patients with diabetes after moderate loss in kidney functiond even without albuminuria. Diabetes Care (2019) 42(10):1886–94. doi: 10.2337/dc19-0349 31221677

[18] Chen S Zhou Y Chen Y Gu J. Fastp: An ultra-fast all-in-one FASTQ preprocessor. Bioinformatics (2018) 34(17):i884–90. doi: 10.1093/bioinformatics/bty560 PMC6129281 30423086

[19] Li H. Minimap2: Pairwise alignment for nucleotide sequences. Bioinformatics (2018) 34(18):3094–100. doi: 10.1093/bioinformatics/bty191 PMC6137996 29750242

[20] Tischler G Leonard S. Biobambam: Tools for read pair collation based algorithms on BAM files. Source Code Biol Med (2014) 9(1):1–18. doi: 10.1186/1751-0473-9-13

[21] Jiang P Sun K Peng W Cheng SH Ni M Yeung PC. Plasma DNA end-motif profiling as a fragmentomic marker in cancer, pregnancy, and transplantation. Cancer Discovery (2020) 10(5): 664–73. doi: 10.1158/2159-8290.CD-19-0622 32111602

[22] Chan RWY Jiang P Peng X Tamc LS Liao GJW Li EKM. Plasma DNA aberrations in systemic lupus erythematosus revealed by genomic and methylomic sequencing. Proc Natl Acad Sci U.S.A. (2014) 111(49):E5302–11. doi: 10.1073/pnas.1421126111 PMC4267379 25427797

[23] Krzywinski M Schein J Birol I Connors J Gascoyne R Horsman D. Circos: An information aesthetic for comparative genomics. Genome Res (2009) 19(9):1639–45. doi: 10.1101/gr.092759.109 PMC2752132 19541911

[24] Devan P Khare N. An efficient XGBoost–DNN-based classification model for network intrusion detection system. Neural Comput Appl (2020) 32:12499–514. doi: 10.1007/s00521-020-04708-x

[25] Mooney CZ Duval RD. Bootstrapping: A nonparametric approach to statistical inference. J Am Stat Assoc (1994) 133(1):59–68. doi: 10.2307/2290969

[26] Sun K Jiang P Wong AIC Cheng YKY Cheng SH Zhang H. Size-tagged preferred ends in maternal plasma DNA shed light on the production mechanism and show utility in noninvasive prenatal testing. Proc Natl Acad Sci U.S.A. (2018) 115(22):E5106–14. doi: 10.1073/pnas.1804134115 PMC5984542 29760053

[27] Chan RWY Serpas L Ni M Volpi S Hiraki LT Tam LS. Plasma DNA profile associated with DNASE1L3 gene mutations: clinical observations, relationships to nuclease substrate preference, and *in vivo* correction. Am J Hum Genet (2020) 107(5):882–94. doi: 10.1016/j.ajhg.2020.09.006 PMC7674998 33022220

[28] Carithers LJ Moore HM. The genotype-tissue expression (GTEx) project. Biopreserv Biobank (2015) 13( 5):307–8. doi: 10.1089/bio.2015.29031.hmm PMC4692118 26484569

[29] Haroun M El-Sayed MM. Measurement of IgG levels can serve as a biomarker in newly diagnosed diabetic children. J Clin Biochem Nutr (2007) 40(1):56–61. doi: 10.3164/jcbn.40.56 PMC2291473 18437206

