# Supplementary figures and images for: The fragmentomic property of plasma cell-free DNA enables the non-invasive detection of diabetic nephropathy in patients with diabetes mellitus
Source: Front Endocrinol (Lausanne). 2023 Oct 5;14:1164822. doi: 10.3389/fendo.2023.1164822 (PMC10586048; doi:10.3389/fendo.2023.1164822)

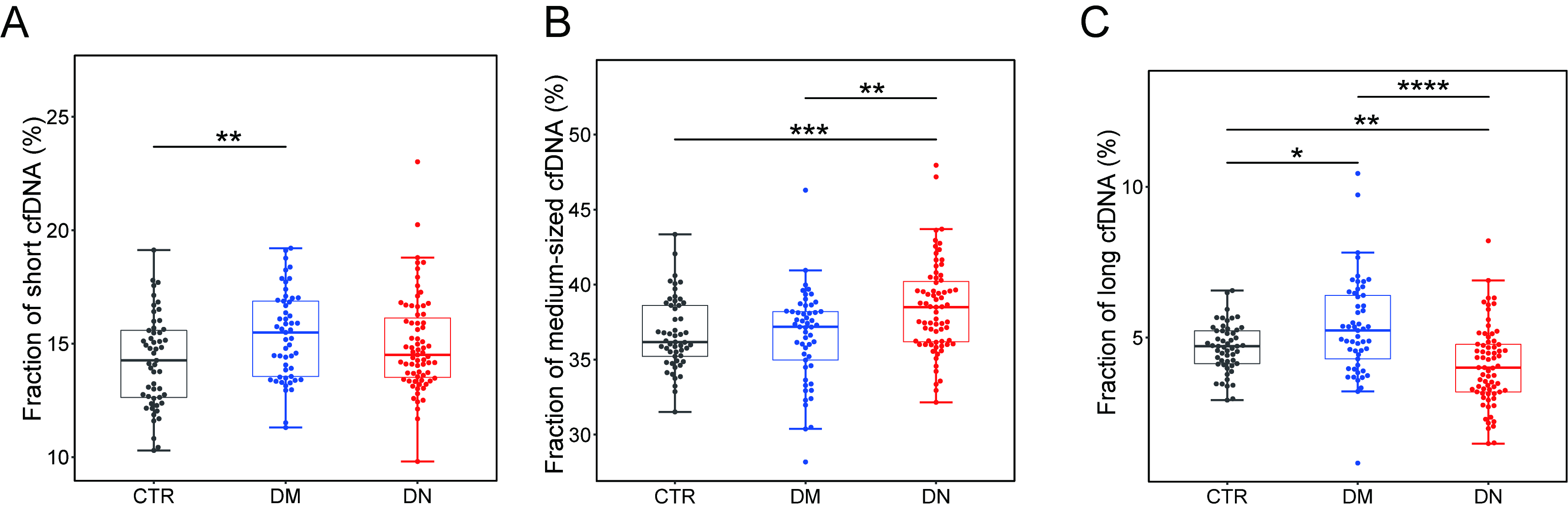

Supplement: Supplementary Figure 1 — (A) Box plot of the fraction of cfDNA with small size, (B) medium size and (C) large size in control, DM and DN subjects. [file Image_1.tif]

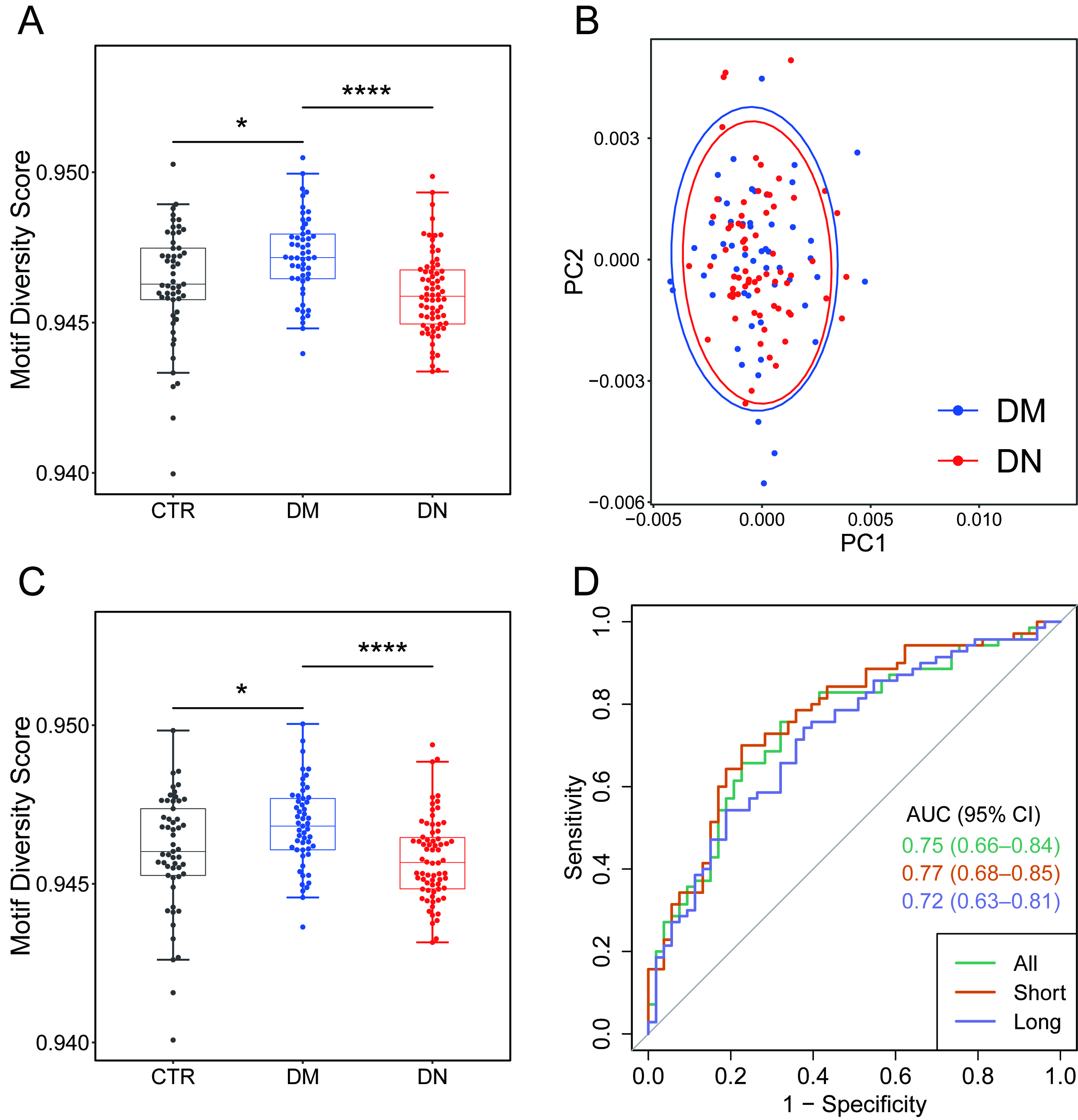

Supplement: Supplementary Figure 2 — Motif diversity score of plasma DNA with different sizes. (A) MDS of plasma DNA with all size. (B) PCA analysis on the frequencies of all 256 motifs of cfDNA without size selection. (C) MDS of plasma DNA with large size. (D) ROC analysis of MDS of plasma DNA with different sizes in the identification of DN patients from DM patients. [file Image_2.tif]

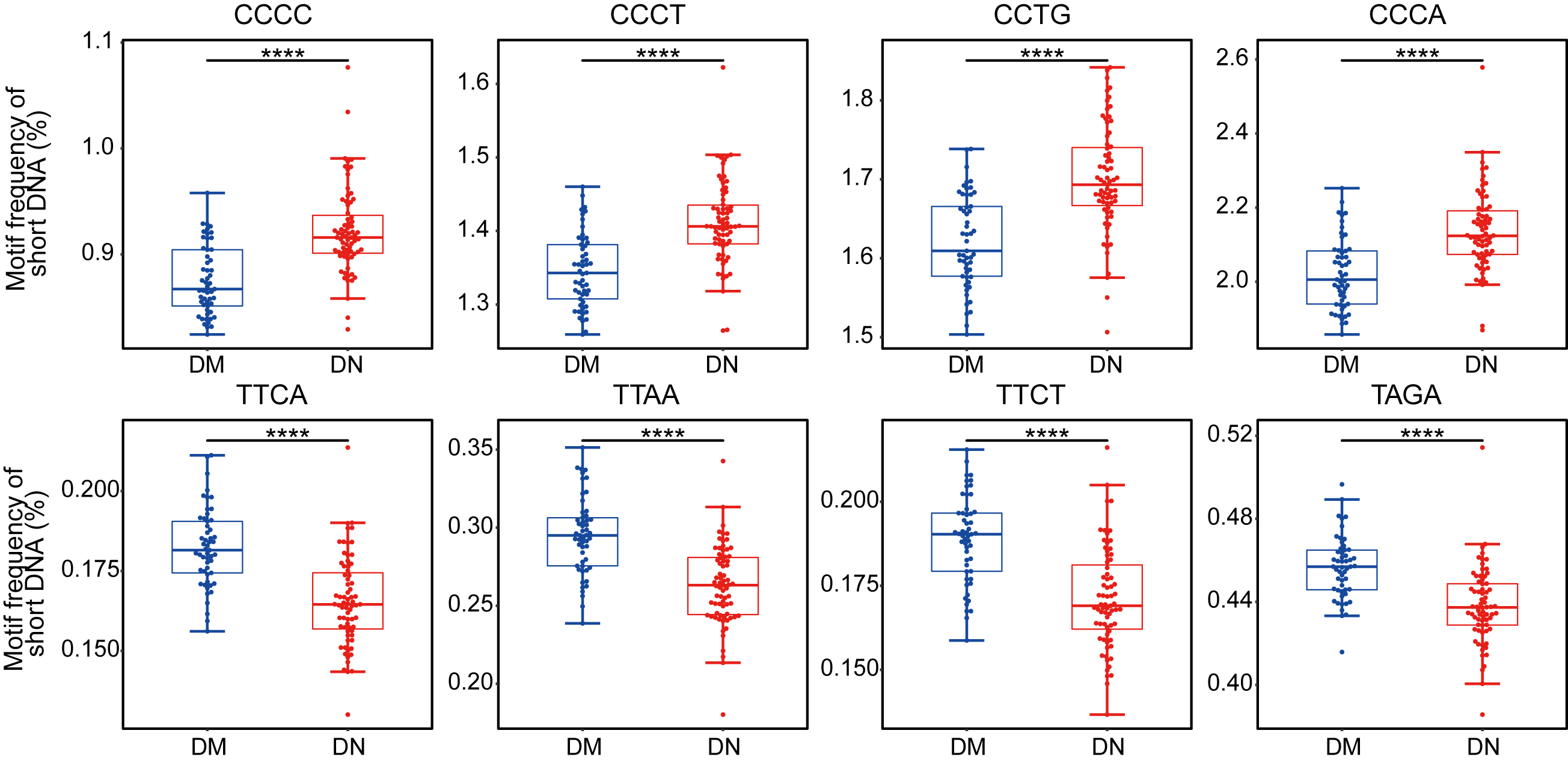

Supplement: Supplementary Figure 3 — Distribution of DN-specific motifs in control, DM and DN groups. [file Image_3.tif]

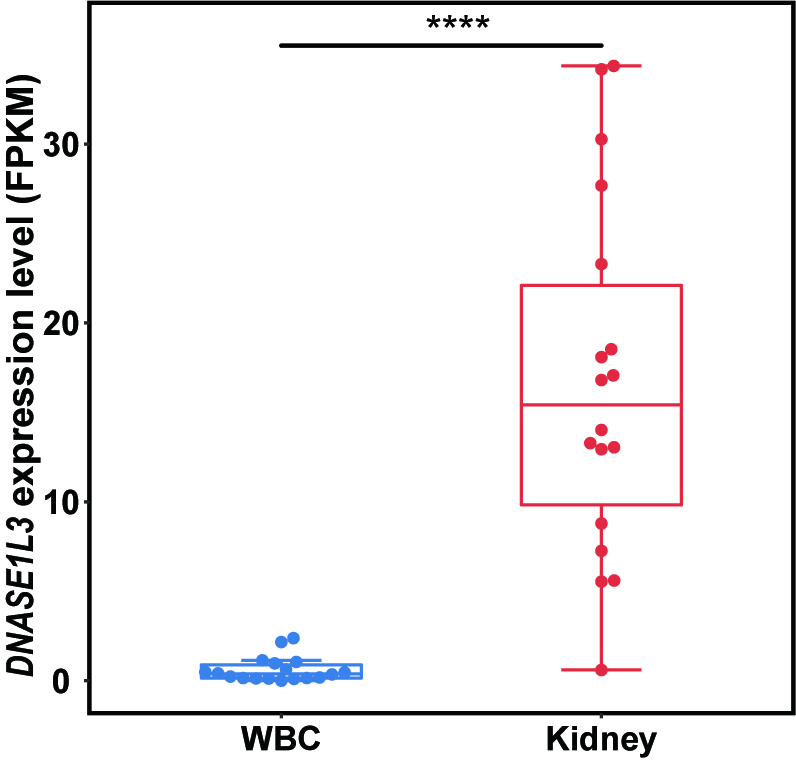

Supplement: Supplementary Figure 4 — Expression levels of DNASE1L3 gene in whole blood cells (WBC) and kidney tissue, which were calculated by the transcriptome data of paired WBC samples and kidney tissue samples of 18 adults from the GTEx database. [file Image_4.tif]

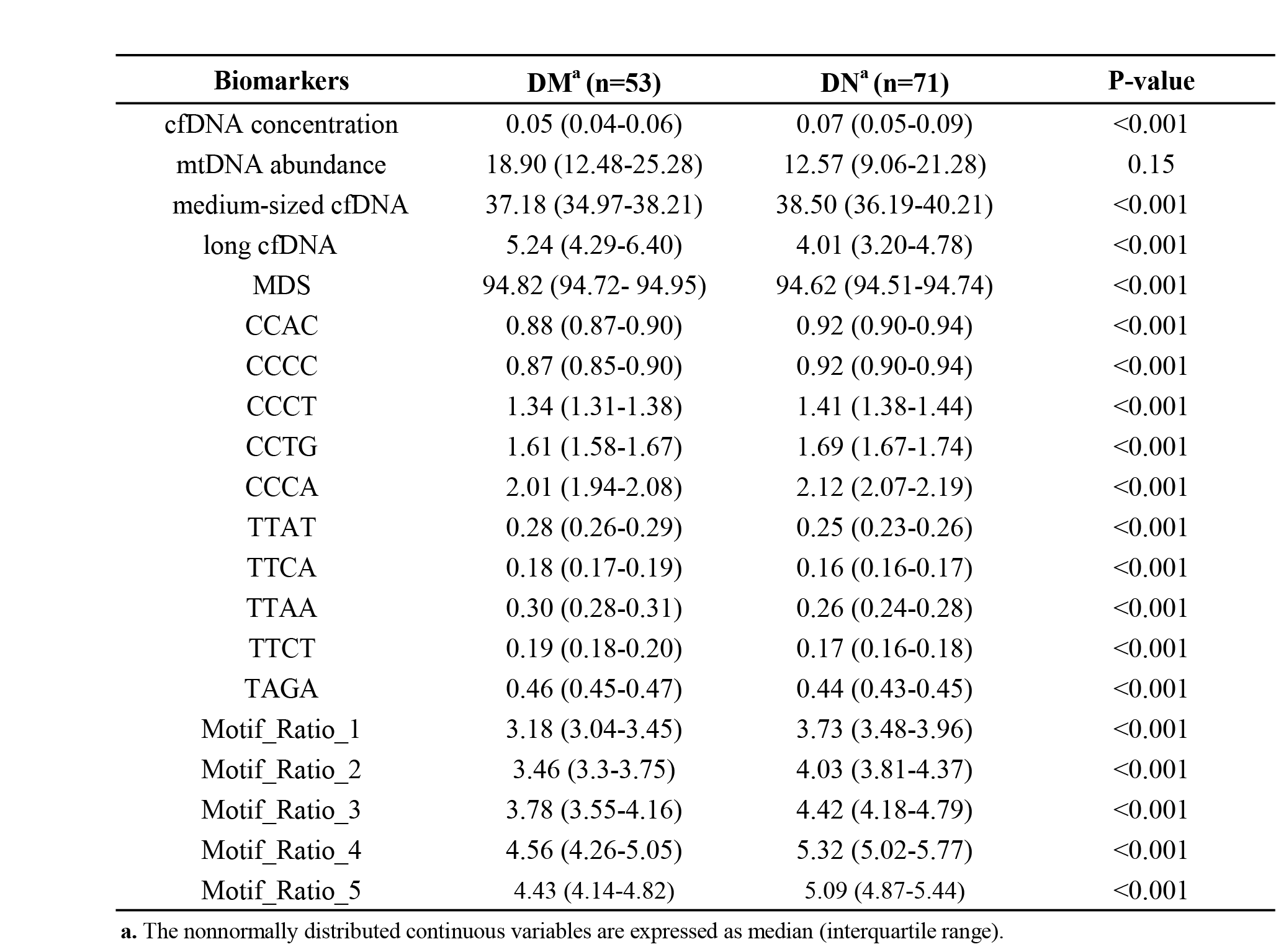
**Supplementary Table 1**

Supplement: Supplementary Table 1 — P-values of the association between cfDNA features and DM/DN outcomes in logistic regression with taking age, gender and BMI as covariates. All the motif related features are based on short cfDNA (<=156bp). [file Table_1.docx]

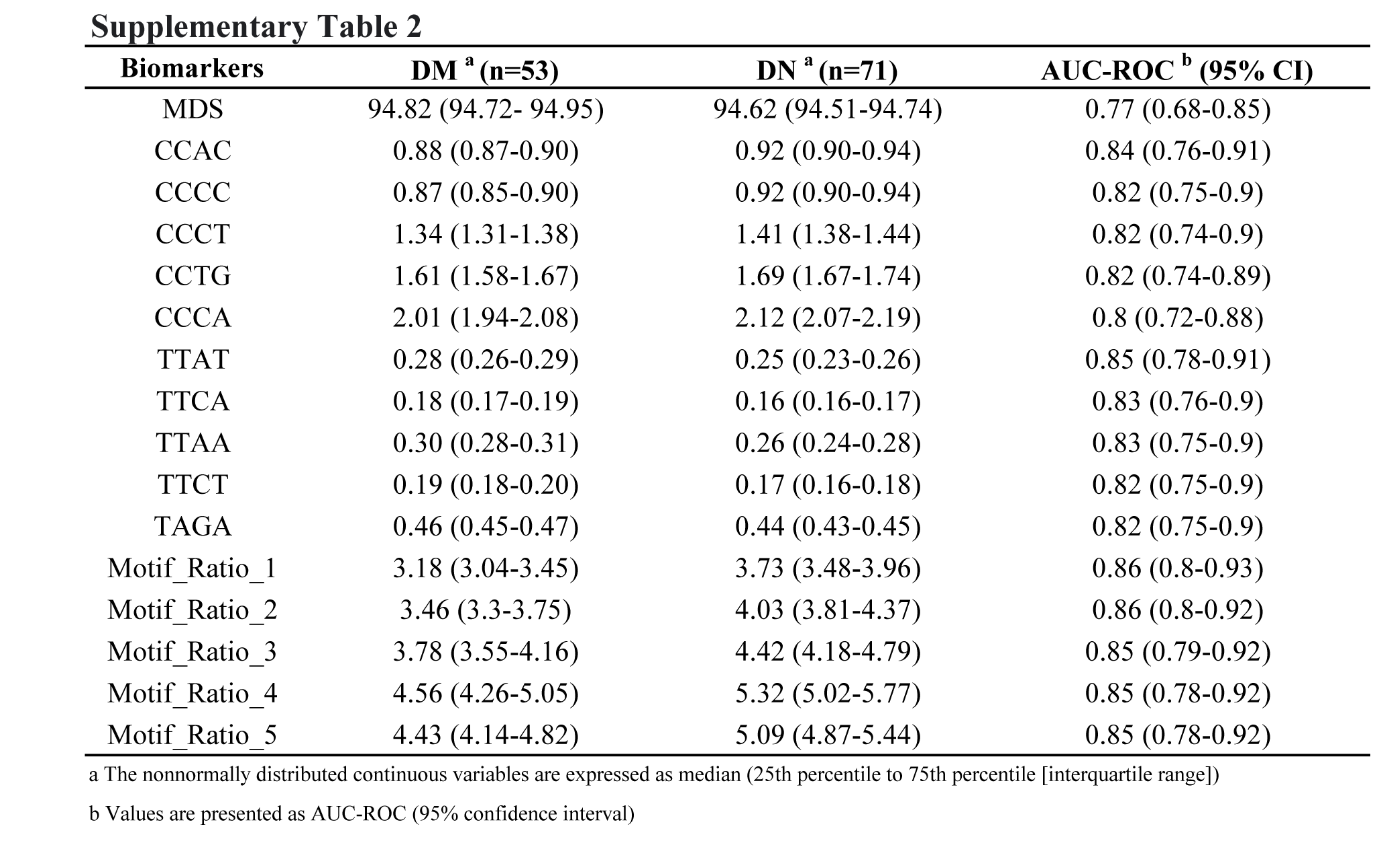
**Supplementary Table 2**

Supplement: Supplementary Table 2 — AUC values of all informative motif features in the determination of DN and DM patients. [file Table_2.docx]
